# Evaluation of the Webler-Brown model for estimating tetrachloroethylene exposure from vinyl-lined asbestos-cement pipes

**DOI:** 10.1186/1476-069X-7-24

**Published:** 2008-06-02

**Authors:** Lisa A Spence, Ann Aschengrau, Lisa E Gallagher, Thomas F Webster, Timothy C Heeren, David M Ozonoff

**Affiliations:** 1 Department of Environmental Health, Boston University School of Public Health, Boston, MA, USA; 2 Department of Epidemiology, Boston University School of Public Health, Boston, MA, USA; 3 Department of Biostatistics, Boston University School of Public Health, Boston, MA, USA

## Abstract

**Background:**

From May 1968 through March 1980, vinyl-lined asbestos-cement (VL/AC) water distribution pipes were installed in New England to avoid taste and odor problems associated with asbestos-cement pipes. The vinyl resin was applied to the inner pipe surface in a solution of tetrachloroethylene (perchloroethylene, PCE). Substantial amounts of PCE remained in the liner and subsequently leached into public drinking water supplies.

**Methods:**

Once aware of the leaching problem and prior to remediation (April-November 1980), Massachusetts regulators collected drinking water samples from VL/AC pipes to determine the extent and severity of the PCE contamination. This study compares newly obtained historical records of PCE concentrations in water samples (n = 88) with concentrations estimated using an exposure model employed in epidemiologic studies on the cancer risk associated with PCE-contaminated drinking water. The exposure model was developed by Webler and Brown to estimate the mass of PCE delivered to subjects' residences.

**Results:**

The mean and median measured PCE concentrations in the water samples were 66 and 0.5 μg/L, respectively, and the range extended from non-detectable to 2432 μg/L. The model-generated concentration estimates and water sample concentrations were moderately correlated (Spearman rank correlation coefficient = 0.48, p < 0.0001). Correlations were higher in samples taken at taps and spigots vs. hydrants (ρ = 0.84 vs. 0.34), in areas with simple vs. complex geometry (ρ = 0.51 vs. 0.38), and near pipes installed in 1973–1976 vs. other years (ρ = 0.56 vs. 0.42 for 1968–1972 and 0.37 for 1977–1980). Overall, 24% of the variance in measured PCE concentrations was explained by the model-generated concentration estimates (p < 0.0001). Almost half of the water samples had undetectable concentrations of PCE. Undetectable levels were more common in areas with the earliest installed VL/AC pipes, at the beginning and middle of VL/AC pipes, at hydrants, and in complex pipe configurations.

**Conclusion:**

PCE concentration estimates generated using the Webler-Brown model were moderately correlated with measured water concentrations. The present analysis suggests that the exposure assessment process used in prior epidemiological studies could be improved with more accurate characterization of water flow. This study illustrates one method of validating an exposure model in an epidemiological study when historical measurements are not available.

## Background

From May 1968 through March 1980, vinyl-lined asbestos-cement (VL/AC) water pipes were installed in the six New England states to avoid taste and odor problems associated with the asphalt-based lining when the recommended alkalinity level was exceeded [1]. The vinyl lining was applied by manually spraying vinyl resin (Piccotex™) dissolved in a solution of tetrachloroethylene (perchloroethylene, PCE) [1,2]. Although the lined pipes were "cured" by drying for two days prior to delivery, large quantities of PCE remained in the liner and subsequently leached into the public drinking water supplies [1,3].

Approximately 660 miles of VL/AC pipes were installed in Massachusetts; a large proportion was installed in the Cape Cod region to replace existing pipe or extend the water distribution system [1,4]. When the Massachusetts Department of Environmental Protection (DEP) became aware of the problem in early 1980, regulators collected drinking water samples to determine the location and severity of the PCE contamination in affected towns [1,4]. Most areas with elevated PCE concentrations were subsequently flushed with large volumes of water or remediated by continuously bleeding the water lines until levels fell below the 1980 Suggested Action Guide (SAG) of 40 μg/L. This SAG was derived from the Environmental Protection Agency (EPA) Suggested No Adverse Response Level (20 μg/L) and assumed that "the problem was not a long-term one" [4]. In some areas, configurations of dead-end pipes were changed, or affected pipes were replaced.

Two years after the PCE contamination was discovered, the Massachusetts Cancer Registry began operations to monitor cancer incidence in the State, and its initial data reported elevated cancer incidence rates in the Cape Cod region [5]. In response to public concern, we conducted a series of population-based case-control studies on cancer risk associated with exposure to air and water pollution, including PCE-contaminated drinking water [6-9]. We estimated cumulative PCE exposure for these investigations using a model developed by Webler and Brown [10]. Webler and Brown's cumulative PCE exposure estimate, which they called the Relative Delivered Dose (RDD), was constructed to be roughly proportional to the mass of PCE that entered a home over a specific time period. RDD calculations used the rate at which PCE leached from the vinyl pipe liner, the surface area of the interior of the pipe, and the water use, or loading, along VL/AC pipe. Various physical factors, thought to have a roughly linear effect on the estimate, were removed making the RDD a proportional exposure metric [10].

This analysis compares PCE concentrations in historical pre-remediation drinking water samples with PCE concentrations estimated using the Webler-Brown model. The objectives were to compare the exposure assessment approach used for the epidemiologic studies with independently measured historical data, and to identify characteristics of the water distribution system, exposure estimation process, and water sampling procedure that affected the correlation between the measured and estimated concentrations.

## Methods

### PCE concentrations in historical drinking water samples

Historical Massachusetts DEP records were reviewed to obtain PCE concentrations in drinking water samples collected in 1980. Sample records were collected for nine Massachusetts towns with VL/AC water distribution pipe (Barnstable, Bourne, Brewster, Chatham, Falmouth, Provincetown, Sandwich, Plymouth, and Wareham). The first seven of these towns were selected because they comprise the geographic site of prior epidemiologic studies. The last two towns were included because they were adjacent to Cape Cod and had a large number of samples available with appropriate documentation.

No written protocol for water sampling was found in DEP files. Additionally, no written records were identified describing the laboratory analysis procedures. The likely equipment used to analyze these samples was a gas chromatograph using heated static head space analysis with a packed column and a Hall electrolytic conductivity detector (Personal communication, Oscar Pancorbo, Director Lawrence Experiment Station, April 2004). This is consistent with various reports [11-13], including a 1980 DEP memorandum stating that "a rapid but non-approved method of analysis based on head-space technology is being employed by the organic analysis section....for measurement of tetrachloroethylene in drinking water." This memorandum suggested that the EPA regarded this as a qualitative, not quantitative, method. Use of head space decreased the analysis time in comparison to purge and trap methods, allowing DEP to analyze samples rapidly.

For the current study, it was necessary to select water samples taken before remediation began [14]. We initially identified 112 sample results from the nine towns. We excluded ten samples taken at locations with no adjacent or upstream VL/AC pipes because these locations provided no opportunity for exposure; one sample taken at a location with insufficient data to estimate the PCE concentration; one sample not analyzed at the DEP laboratory; eleven samples taken at locations with no water flow because no houses were in the area (the model assumes water is not stagnant); and one sample taken at a location with unusually high water flow not seen in residential areas of our prior epidemiologic studies. The exclusions were done prior to analyzing the data. There remained 88 samples for the current analysis. We extracted the following information on each water sample: collection date, location (street and town), water fixture sampled (tap, spigot, or hydrant), and the name of the DEP employee collecting the sample.

### Estimated PCE concentrations

The Webler and Brown model developed for our prior epidemiologic studies [6-9] produces a cumulative exposure measure (RDD) [10]. We adapted the Webler-Brown model for the current analysis by generating point concentration (PC) estimates more appropriate for comparison with measured PCE concentrations in water samples taken on a single occasion. The adapted model specifies the RDD estimate retaining all constants but without integrating over time:

(1) $$PC_{i}(t)=\int_{x=0}^{i}\frac{C_{0}e^{\frac{-(T)}{r}}\pi D_{x}}{rQ_{x}}dx.$$

In this equation, *C*_0 _is the initial amount of PCE per unit surface area (μg/m^2^), *T *is the lapsed time from pipe installation (*t*_*s*_) to the year that the water sample was taken (*t*), *r *is the diffusion rate constant (years), *D*_*x *_is the pipe diameter (meters), and *Q*_*x *_is the water flow rate (liters/year). Thus, *C*_0_*e *^-(*T*/*r*) ^is the amount of initial PCE remaining in the liner after time *T *with first order (exponential) decay [2]. Integration is along a pipe (*dx*) to the location of interest, *i*.

The integral is approximated by summing discrete pipe segments that were designated to implement the model. Each segment ends at a node, defined as the end point of a segment. The model is developed around these segments (*s*) and nodes and *dx *becomes the pipe segment length (*L*_*s*_).

(2) $$PC_{i}(t)=\sum_{s=1}^{i}\frac{C_{0s}e^{\frac{-(t-t_{s})}{r}}\pi D_{s}L_{s}}{rQ_{s-1}}$$

Water drawn along a segment is considered removed at the segment node. Thus, the water flow in segment *s *is *Q*_*s*-1._. Equation (2) gives the estimated micrograms of PCE per liter drinking water at location *i *at time *t *– the quantity that was compared to the measured PCE drinking water concentrations in the current study. See Appendix A for a derivation of Equation (2).

Exposure assessments for the present study were conducted by two individuals using Equation (2) and following procedures developed in our prior epidemiological studies [6-9]. Water supply distribution maps, including the locations of all VL/AC pipes, pipe diameters and installation years, were obtained from the Massachusetts DEP and town water departments. Parcel maps indicating the locations of residences were provided by town officials. Each historical water sampling point was located on the distribution network, and a schematic was made depicting the water flow at each sample location.

The quantity of water flow (evaluated as loads) was estimated as the number of parcels at and beyond a VL/AC pipe segment. Each parcel was assumed to represent one single-family home, the most common type of residence in the geographic area. Water flow was determined after consulting with water department officials and inspecting features of the distribution network, including pipe diameters and locations of wells and pumping stations. Flow assessments were conducted using simplifying assumptions outlined by Webler and Brown [10]. These simplifying assumptions were as follows: (1) water flows along the most direct route from larger-diameter pipes to smaller ones, (2) water flow is constant over time, (3) all parcels draw the same quantity of water, and (4) water demand beyond a given neighborhood has a negligible impact on flow direction and volume.

### Data analysis

We conducted descriptive analyses to characterize the measured PCE concentrations among all samples combined and among samples stratified according to characteristics of the water distribution system, and other factors that may affect exposure estimation and water sampling procedure. Samples with undetectable PCE levels were assigned a value of 0.25 μg/L, one-half the laboratory detection limit of 0.5 μg/L [15]. Substitutions with zero, the detection limit (0.5 μg/L), and the detection limit divided by the square-root of two (0.35 μg/L) for samples where PCE was not detected (ND) were also conducted [15,16] and gave similar results. A logistic regression analysis was carried out to determine characteristics associated with undetectable PCE concentrations in the water samples.

Measured drinking water concentrations were compared to estimated concentrations using Spearman rank correlation coefficients. A linear regression model was also used to quantify the proportion of the variance in the measured concentrations explained by the modeled estimates. The natural logarithm (ln) of measured and estimated PCE concentrations was used in the regression model because the data were skewed with a long upper tail. p-values were used to describe the statistical stability of all parameters.

Comparisons were made among all samples combined, samples stratified according to sampling and location characteristics, and samples with detectable PCE levels. Stratification characteristics were 'town,' 'sampling personnel,' 'season of sampling,' 'water fixture sampled,' 'pipe installation year,' 'complexity of pipe configuration,' 'position along pipe,' 'magnitude of water flow,' and 'housing density' (see below for description of these variables). Lastly, because our prior epidemiologic analyses [6-9] categorized subjects according to exposure percentile (*e.g*., > 50^th ^percentile), we examined the measured and estimated PCE concentrations in percentile categories and evaluated the sensitivity and specificity of the estimated concentrations in correctly classifying the lower 50^th ^percentile, upper 50^th ^percentile, and upper 75^th ^percentile of PCE concentrations measured in the water samples.

#### Town

This variable characterized possible differences in sampling protocols and water distribution characteristics. Towns included Barnstable (n = 7 samples), Bourne (n = 16), Brewster (n = 7), Chatham (n = 6), Falmouth (n = 6), Provincetown (n = 5), Sandwich (n = 9), Plymouth (n = 25), and Wareham (n = 7).

#### Sampling personnel

This variable captured undocumented differences in the selection of the sampling location and procedure by the person conducting the sampling. Almost all of the samples were collected by two DEP employees (n = 69 for Sampler 1, n = 18 for Sampler 2; n = 1 for Sampler 3).

#### Sampling season

Most samples were collected in April 1980 (n = 71) shortly after the PCE contamination was publicized. However, some samples were collected in May and the following autumn. As a rough measure of seasonal changes in water temperature, season of sample collection was evaluated. 'Spring' included samples collected in April and May (n = 74) and 'autumn' included samples collected in September and November (n = 14). We hypothesized that PCE leaching rates would be higher in the fall when water temperatures are higher.

#### Water fixture

This variable captured unknown sampling conditions, including flow intensity during sampling and aeration both before and during sampling. Three types of fixtures were sampled: taps (n = 3), spigots (n = 7), and fire hydrants (n = 18). The former two were combined and represent low flow intensity while the latter represents variable flow intensity. Hydrants were also likely to have long-standing air pockets into which PCE could volatilize and were supplied by spur segments from the main VL/AC pipe that may have contained stagnant water. The kind of the collection point was not specified for 60 samples; these samples were treated as a separate category.

#### Pipe installation year

VL/AC pipes were installed on Cape Cod from May 1968 through March 1980. Because PCE drinking water levels decreased exponentially following pipe installation, we characterized water samples according to the installation year of the closest VL/AC pipe. Three categories of roughly equal duration were used: 1968–1972 (n = 23), 1973–1976 (n = 32), and 1977–1980 (n = 33).

#### Complexity of pipe configuration

This variable captured the difficulty in determining the direction of water flow by classifying the pipe configuration in the immediate vicinity of the water sample as 'simple' or 'complex.' The 'simple' category described dead-end pipes that were either directly off a major pipe or close to a water source, thereby ensuring that there was only one possible flow direction, and facilitating the flow rate determination. The 'complex' category described areas with multiple possible flow directions and where it was difficult to determine the area of water demand.

#### Position along pipe

This variable described the proximity of a sample location to the end of the pipe. An 'end' position was designated for locations within the last 25% of VL/AC pipe (n = 44). Locations within the first 75% of VL/AC pipe were designated as 'beginning/middle' (n = 44).

#### Magnitude of water flow

This variable categorized the amount of water flowing past the sampling location into 'high,' 'medium,' and 'low' based on the number of loads around a sampling location. The magnitude of flow was considered 'high' when more than 19 homes were served just downstream by the water pipe at the sample location (n = 19). It was considered 'medium' when 3–19 homes were served by the water pipe (n = 33), and 'low' when 1 or 2 homes were served by the water pipe (n = 36). These cutoffs correspond to the tertiles of the loading distribution.

#### Housing density

Because parcel maps provided by town officials dated from 1988 or later, there was an eight to twenty year gap between VL/AC pipe installation and parcel data used for the model-estimated concentrations. Thus, it is likely that our earlier epidemiological studies [6-9] overestimated the number of homes and water flow, and subsequently underestimated RDDs in areas with recent home construction. Because some water samples in the current analysis were taken in areas with recent home construction, we reviewed town assessor's files for home construction years and DEP files for home water service connection dates, and created a variable to describe this situation. We designated water samples in areas with 'overestimated' housing density if 90% of parcels near the sample location were undeveloped or had homes built more than a year after the VL/AC pipe was installed (n = 25). The remaining sampling locations were designated as not being affected by overestimated housing density (n = 63).

Lastly, we conducted quantitative sensitivity analyses to determine the impact of measurement error on the correlation coefficients. We considered two sources of error based on our knowledge of the water distribution systems and laboratory analysis. The first source stemmed from using a single water sample (taken at a single time point) to characterize fluctuating PCE levels. In reality, up to two-fold fluctuations in the water concentrations were seen in a PCE sampling study that measured concentrations at the same location and time on two consecutive days [12]. The second source of error arose from the use of the head space laboratory analysis which, according to a DEP analysis, underestimated the PCE concentrations in the water samples by as much as 80%. Each source of error was considered in separate sensitivity analyses using a matrix of 500 adjustment factors randomly generated from a uniform distribution. The mean, standard deviation and range were calculated from the resulting distributions of Spearman correlation coefficients.

## Results

The mean and median measured PCE concentrations were 66 μg/L and 0.5, respectively, for all 88 eligible samples combined. Individual sample concentrations ranged from undetectable to 2432 μg/L (Table 1). Even though this analysis was limited to samples taken at a VL/AC pipe, 49% of the samples had undetectable PCE levels (Table 1). The distribution of PCE concentrations was skewed with a long upper tail. The maximum detected PCE concentration (2432 μg/L) was more than three times greater than the next highest concentration.

**Table 1:** PCE concentrations (ug/L) measured in water samples according to characteristics of sampling location and methods

	No. of Samples	No. With ND^a ^Level	Percent With ND^a ^Level	Mean^b^	Median	75^th ^Percentile	Range
All Samples	88	43	49	66	0.5	32	ND-2432
							
According to:							
Complexity of pipe configuration^c^							
Simple	31	13	42	73	20	50	ND-780
Complex	57	30	53	63	ND	22	ND-2432
Magnitude of flow^c^							
High (> 19 homes)	19	11	58	10	ND	20	ND-59
Medium (3–19 homes)	33	22	67	18	ND	20	ND-190
Low (< = 2 homes)	36	10	28	141	18	57	ND-2432
Position along pipe^c^							
End	44	16	36	56	12	37	ND-780
Beginning/Middle	44	27	61	77	ND	25	ND-2432
Overestimated housing density^c^							
Yes	25	11	44	182	6.6	38	ND-2432
No	63	32	51	20	ND	28	ND-235
Season sampled^c^							
Spring	74	42	57	77	ND	38	ND-2432
Autumn	14	1	7	7.7	4.6	11	ND-44
Water fixture sampled^c^							
Tap or spigot	10	3	30	302	32	190	ND-2432
Hydrant	18	7	39	26	21	35	ND-92
Unknown	60	33	55	39	ND	18	ND-780
Sampling personnel^c^							
Sampler 1	69	33	48	38	0.5	31	ND-780
Sampler 2	18	10	56	43	ND	28	ND-350
Sampler 3	1	0	0	2432	---^d^	---^d^	---^d^
Town^c^							
Barnstable	7	7	100	ND	ND	ND	ND-ND
Bourne	16	10	63	57	ND	26	ND-540
Brewster	7	2	29	140	32	100	ND-780
Chatham	6	5	83	4.9	ND	ND	ND-28
Falmouth	6	1	17	47	53	62	ND-75
Provincetown	5	5	100	ND	ND	ND	ND-ND
Sandwich	9	3	33	36	22	59	ND-92
Plymouth	25	7	28	128	3.1	13	ND-2432
Wareham	7	3	43	15	20	28	ND-35
Pipe installation year^c^							
1968–1972	23	16	70	38	ND	1	ND-780
1973–1976	32	15	47	18	4.6	25	ND-100
1977–1980	33	12	36	134	22	59	ND-2432

^a ^ND – Not Detected. The detection limit was 0.5 μg/L.

^b ^Means were calculated using 0.25 μg/L (half the detection limit) for ND samples.

^c ^See text for definitions and further description.

^d ^Analyses were not conducted.

The highest median concentrations were observed in Brewster (32 μg/L) and Falmouth (53 μg/L), and in samples collected in areas with simple pipe configuration (median = 20 μg/L), from taps and spigots (median = 32 μg/L); and along the most recently installed (1977–1980) VL/AC pipes (median = 22 μg/L) (Table 1).

Conversely, undetectable levels were reported in all or nearly all samples collected in Barnstable (100%), Provincetown (100%), and Chatham (83%) (Table 1). In addition, undetectable levels were more common in samples collected from the earliest installed (1968–1972) VL/AC pipe (70%), where water flow was medium or high (67% and 58%), and at the beginning/middle of VL/AC pipe (61%). Because these characteristics were correlated, we conducted a multiple logistic regression analysis to determine which factors predicted undetectable PCE levels, while controlling for the other factors. Undetectable PCE levels were more common in areas with the earliest installed VL/AC pipes (adjusted OR: 8.4), where flow was high (adjusted OR: 6.3), at the beginning and middle of VL/AC pipes (adjusted OR: 2.1), and at unknown sampling locations (adjusted OR: 3.7).

**Figure 1:**
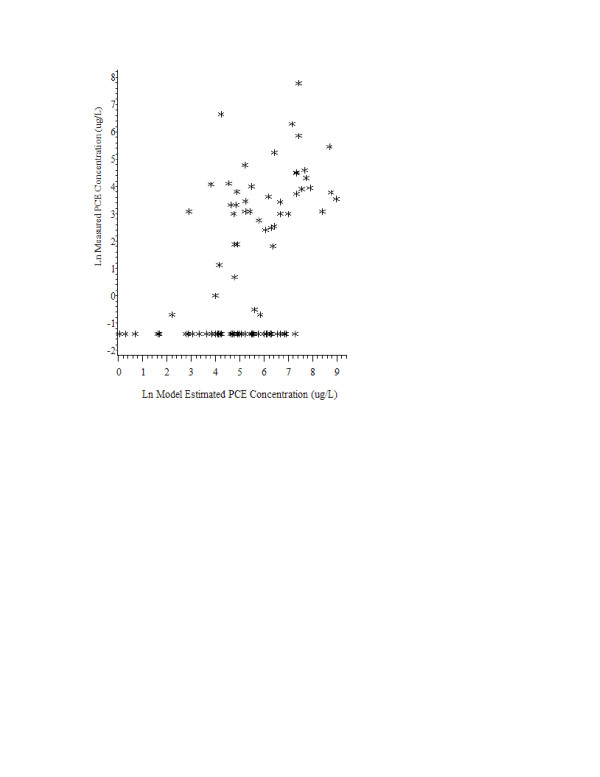
Log_e _measured PCE verses log_e _model estimated PCE concentrations (ug/L).

The relationship between measured and estimated PCE concentrations is shown in Figure 1. The horizontal line of points along the bottom of the graph represents samples with no detectable concentration of PCE. Overall, there was a moderate level of correlation between PCE concentrations in the water samples and point concentration estimates derived from the adapted Webler and Brown model (Spearman rank correlation coefficient (ρ) = 0.48, p < 0.0001, Table 2). According to the regression analysis, 24% of the variance in measured PCE concentrations was explained by modeled concentrations (p < 0.0001).

**Table 2:** Correlation coefficients between measured PCE concentrations in water samples and model-generated estimates

	No. of Samples	Spearman Correlation Coefficient	P-Value
All Samples	88	0.48	< 0.001
			
According to:			
Complexity of pipe configuration^a^			
Simple	31	0.51	0.003
Complex	57	0.38	0.003
			
Magnitude of flow^a^			
High (> 19 homes)	19	0.37	0.1
Medium (3–19 homes)	33	0.30	0.09
Low (< = 2 homes)	36	0.54	0.0007
			
Position along pipe^a^			
End	44	0.44	0.003
Beginning/Middle	44	0.44	0.003
			
Overestimated housing density^a^			
Yes	25	0.52	0.008
No	63	0.47	0.0001
			
Season sampled^a^			
Spring	74	0.50	< 0.0001
Autumn	14	0.58	0.03
			
Water fixture sampled^a^			
Tap or spigot	10	0.84	0.002
Hydrant	18	0.34	0.2
Unknown	60	0.38	0.003
			
Sampling personnel^a^			
Sampler 1	69	0.45	0.0001
Sampler 2	18	0.57	0.01
Sampler 3	1	---^b^	---^b^
			
Town^a^			
Barnstable	7	---^c^	---^c^
Bourne	16	0.53	0.03
Brewster	7	0.018	1.0
Chatham	6	0.39	0.4
Falmouth	6	0.49	0.3
Provincetown	5	---^c^	---^c^
Sandwich	9	0.53	0.1
Plymouth	25	0.56	0.003
Wareham	7	0.48	0.3
			
Pipe installation year^a^			
1968–1972	23	0.42	0.05
1973–1976	32	0.56	0.0008
1977–1980	33	0.37	0.03

^a ^See text for definitions and further description.

^b ^Analyses were not conducted because of the small sample size.

^c ^Analyses were not conducted because all water samples had undetectable PCE levels.

Results of the quantitative sensitivity analysis indicated that the correlation level was robust. The mean Spearman correlation coefficient between the randomly adjusted PCE concentrations in the water samples and point concentration estimates from the model was 0.44 (σ = 0.04, p < 0.0001), and the range extended from 0.29 to 0.53.

The correlation varied according to sampling characteristics. Correlations were higher among samples collected at taps and spigots vs. hydrants (ρ = 0.84 vs. 0.34), and by Sampler 2 vs. Sampler 1 (ρ = 0.57 vs. 0.45). Correlations also varied by factors that may affect exposure estimation: areas with simple vs. complex geometry (ρ = 0.51 vs. 0.38), at low vs. medium and high flow locations (ρ = 0.54 vs. 0.30 and 0.37), and near pipes installed in 1973–1976 vs. earlier and later years (ρ = 0.56 vs. 0.42 for 1968–1972 and 0.37 for 1977–1980). The correlation also varied considerably by town; it was highest in Plymouth (ρ = 0.56) and lowest in Brewster (ρ = 0.02). The lack of correlation in Brewster stemmed from the highest measured PCE concentration in Brewster (780 μg/L) that was predicted to be the town's lowest concentration (82 μg/L). When this location was excluded, the Spearman correlation coefficient for Brewster was 0.64. There was little difference in the correlation according to housing density estimates (ρ = 0.52 vs. 0.47), pipe position (ρ = 0.44 vs. 0.44), and season (ρ = 0.58 vs. 0.50).

When analyses were limited to samples with detectable PCE levels, the highest median concentrations were observed among samples collected in areas with simple pipe configuration (median = 40 μg/L), from taps and spigots (median = 100 μg/L); and along the most recently installed (1977–1980) VL/AC pipes (median = 45 μg/L). This pattern is similar to the entire sample. However, the Spearman correlation coefficient fell to 0.41 (p = 0.005), and the amount of explained variance fell to 19% when the analysis was restricted to these samples. The data were too sparse to stratify the correlations according to the water distribution, exposure estimation, and sampling characteristics.

Table 3 presents the relationship between the modeled and measured PCE concentrations in the percentile categories used in our prior epidemiological studies (Table 3). The cutoffs for the 50^th ^and 75^th ^percentile categories were 207 ug/L and 657 ug/L, respectively, among the modeled PCE concentrations while they were 0.5 ug/L and 32 ug/L among the measured PCE concentrations. The large difference in concentration distributions stems mainly from the sizeable number of undetectable levels in the measured samples (Figure 1). The percentile categories of the model estimated and measured concentrations were identical in 54.5% of samples. A balanced pattern of discordance was seen among the remaining samples with 23.9% of model estimated concentrations in higher percentile categories than measured concentrations, and 21.6% of modeled estimated concentrations in lower percentile categories than measured concentrations. The sensitivities of the model estimated concentrations for correctly classifying the upper 50^th ^and upper 75^th ^percentiles of the measured PCE concentrations were 63% and 59%, respectively. The corresponding specificities were 62% and 86%.

**Table 3:** Number of samples according to percentile categories^a ^of measured and modeled PCE concentrations

	Measured PCE concentration				
		≤ 50^th ^percentile	> 50–75^th ^percentile	> 75^th ^percentile	Total

Model estimated PCE concentration	≤ 50^th ^percentile	28	10	6	44
	> 50–75^th ^percentile	12	7	3	22
	> 75^th ^percentile	5	4	13	22

	Total	45	21	22	88

^a ^207 μg/L and 657 ug/L were the 50^th ^and 75^th ^percentile modeled PCE concentrations, respectively. 0.5 μg/L and 32 ug/L were the 50^th ^and 75^th ^percentile measured PCE concentrations, respectively.

## Discussion

Our study found a moderate, statistically significant correlation between measured and estimated PCE concentrations (Spearman correlation coefficient ρ = 0.48, p < 0.0001). The correlation varied across characteristics of the water sampling procedures; correlations were higher among samples taken at taps and spigots compared to hydrants. Correlations also varied across factors that we hypothesized might affect the accuracy of the estimation procedure; correlations were higher in areas with simple geometry, low flow, and near pipes installed in the earlier years. In contrast, the lowest correlations were observed in areas with complex geometry, and near pipes installed in the most recent years. About 55% of the model estimated and measured concentrations were in identical percentile categories when the data were examined in groupings used in our prior epidemiological studies.

Even though the current analysis was limited to samples taken at VL/AC pipes, only 51% of the samples had detectable PCE levels. The correlation between estimated and measured PCE concentrations was 0.41 (p = 0.005) among these samples. Undetectable PCE levels were more common in areas with the earliest installed VL/AC pipes, at the beginning and middle of VL/AC pipes, at hydrants, in complex pipe configurations, and where housing density estimates were considered more accurate.

These results suggest that (1) the sampling procedures and analytical methods affected the accuracy of the measured PCE concentrations, and (2) the exposure model and assessment process had inaccuracies that depended on the characteristics of the sampling location that, in turn, affected the correlation between measured and predicted concentrations.

### Inaccuracies in Measured PCE Concentrations

The historical water samples to measure the PCE concentrations were not collected with the goal of validating the exposure model used in our epidemiologic study, but "to determine quickly the extent and severity" of a public health problem in 1980 [4]. DEP focused on locations "where lined VL/AC pipe was in use [and] the pipe was installed in ... dead-end or low flow locations" [4]. Thus, the measured PCE concentrations should be considered an "alloyed gold standard," a term used by Wacholder *et al*. [17] to describe error-prone reference procedures used in validation studies [18].

Savitz has suggested that a spot measurement is not a gold standard for long-term, cumulative exposures, despite "all the appearances of accuracy" because it reflects "only a single point in time in a fluctuating system..." [19]. In our case, Yuskus characterized fluctuations in PCE point concentrations in a VL/AC pipe in a 24-hour sampling study in one Cape Cod town and found that measured concentrations at the same location and time on two consecutive days differed about two-fold [12]. To the best of our knowledge, the water samples in our study were collected during regular working hours. Any short-term fluctuations were not reflected in our model and so likely reduced the correlation between the measured and estimated concentrations.

Moreover, the laboratory's use of head space analysis may have inconsistently reduced PCE recoveries, thereby reducing the correlation between the measured and estimated concentrations. The head space laboratory analysis, which was done to facilitate timely analysis of hundreds of drinking water samples, relies on the tendency of PCE to volatilize out of water into air. In contrast, the more accurate purge and trap method removes PCE from water by purging the water with an inert gas and then trapping the PCE on a solid sorbent. Duplicate sample analyses conducted by the DEP laboratory suggested that the head space analysis inconsistently underestimated the PCE concentrations. In one set of analyses, the concentration observed using head space analysis was only 20% of that using the purge and trap method (38 and 205 μg/L, respectively), while the concentrations were similar in the second set (160 and 150 μg/L, respectively).

A large proportion of samples had missing data on the water fixture that was sampled. If the remaining results are unbiased, the data suggest that sampling from hydrants may also have introduced error from increased aeration. Hydrant samples had lower measured concentrations (median = 21 ug/L) and one of the lowest correlations (ρ = 0.34, p = 0.2), while tap and spigot samples had the highest measured PCE concentrations (median = 32 ug/L) and the highest correlation with the estimated concentrations (ρ = 0.84, p = 0.002). High flow fixtures such as fire hydrants likely introduced air into water samples, thereby reducing the amount of PCE remaining in the water by air stripping. Fire hydrants may also have had a head space of air, with the loss of PCE along the interface between the water and air. In contrast, taps and spigots were capable of generating the low water flow more suited for characterizing volatile organic compounds, and were less likely to have a high volume head space.

Although all samples were collected along VL/AC pipe, 49% had undetectable PCE levels. In fact, the large difference in concentration distributions stems mainly from the large number of undetectable levels (Figure 1). We believe that the use of head space analysis was partially responsible for these undetectable levels. However, because samples with undetectable PCE levels were seen across a broad range of model-estimated concentrations, it is likely water distribution and sampling characteristics also contributed to the undetectable levels. Our analyses found that undetectable levels were associated with sampling from complex pipe configurations, hydrants or unknown locations, beginning and middle pipe positions, and the early installation years.

### Inaccuracies in the Webler Brown model and its implementation

Our prior studies used the Webler-Brown model to estimate cumulative PCE exposure. This model was specifically developed for epidemiological research and not risk assessment. The model used the rate at which PCE leached from the vinyl liner, the surface area of the interior of the pipe, and the loading along the pipe to calculate the RDD, a measure assumed to be roughly proportional to the mass of PCE that entered a home over a specific time period. Simplifying assumptions about the rate and direction of the water flow were needed to implement the model for our epidemiological studies and the present analysis. These simplifications likely decreased the correlation between the estimated and measured concentrations, particularly in areas with complex pipe configurations because water flow direction and magnitude are less predictable in these settings.

Further, while our assumption that every parcel used water at the same rate and that water was constantly flowing was reasonable given that predominant housing on Cape Cod was a single-family dwelling [20] and few industrial sites were present on Cape Cod during the exposure period [21], this assumption also likely reduced the correlation in areas that had higher water demands from commercial and industrial activities, multi-family dwellings, or lower demands from undeveloped parcels.

Variation in the initial amount of PCE in the pipe or inaccuracy in the diffusion rate constant (*r*) of the Webler-Brown exposure model may also have reduced the correlations in the present study. The model assumes a uniform amount of PCE in the Piccotex™ liner, an even liner thickness, and constant water temperature. In reality, none of these factors were unvarying. For example, Guilmartin *et al*. reported that the thickness of the vinyl liner in VL/AC pipes varied extensively and that the initial amount of PCE in the liner varied due to differences in thickness and drying times [3]. In addition, the diffusion constant was derived from experiments conducted by Demond at 20°C [2], but drinking water temperatures fluctuate seasonally from 11 to 27°C degrees [22]. In contrast, we found evidence to support Demond's finding that the diffusion coefficient decayed exponentially as the pipe aged. The better correlation with early installation years suggests that the slope of exponential decay decreases dramatically with time both in the pipe and the model. Hence, there is less variation and more correlation between the estimated and measured values.

### Other research evaluating exposure to drinking water contaminants

Many studies have evaluated the validity of models to predict trihalomethanes levels in drinking water following treatment with chlorination [e.g., [23,24]]. These models, which were developed to help utilities comply with drinking water regulations, typically include physical characteristics of the water, such as chlorine and organic carbon concentrations, water temperature, and pH. Depending on the setting, these models show good to excellent prediction of measured trihalomethane concentrations; the explained variances range from .37 to .86 [e.g., [23,24]].

Only a few prior studies have, like us, evaluated historical exposure measures developed for epidemiological research. These results of these studies are similar to ours. For example, Freedman et al. evaluated the validity of using nitrate concentrations in public drinking water supplies from a single year to characterize long-term exposure for a case-control study of non-Hodgkins lymphoma and leukemia [25]. The authors compared long-term average nitrate measurements from 1947 through 1975 to recent measurements from 1980, and found a moderate level of overall correlation (Spearman correlation coefficient was 0.54, 95% CI:0.44, 0.63). However, the correlation varied considerably when the data were stratified by the subject's length of residence: the correlation coefficient ranged from 0.17 among subjects with less than 10 years at their 1980 residence to 0.70 among subjects with more than 33 years at their 1980 address. The authors posited that the higher correlation among subjects with stable residential histories reflected the elimination of variability from the location of the water source.

In addition, Ayotte et al. evaluated the validity of a logistic regression model to predict the occurrence of arsenic in ground water for historical exposure assessments among subjects in an epidemiological study of bladder cancer [26]. The model, which took into account geologic and anthropogenic sources of arsenic, geochemical processes, and hydrogeologic and land use factors, predicted the probability of arsenic exceeding 5 ug/L in drinking water wells in New England. The model correctly classified 79.8% of the water samples a random validation data set (n = 380); the sensitivity was 37.1% and specificity was 92.5%.

Lastly, Whitaker et al. examined the validity of a stochastic model to predict exposure to disinfection by-products for a study of adverse birth outcomes [27]. The percentile categories of the model estimated and measured total trihalomethane (THM) concentrations were identical in 74.8 – 85.1% of samples (depending on the water supply region), and the sensitivities of the model estimated concentrations for correctly classifying a "high" THM exposure level ranged from 70.2% to 84.5%.

## Conclusion

In summary, the Webler-Brown model generated exposure estimates moderately concordant with historically measured PCE data. While these findings are similar to those from other studies of historic exposures, this evaluation suggests that more accurate water flow characterizations would further improve the correlation with historical water data, acknowledging these data are themselves subject to systematic error. Water pipe distribution models are now available to determine flow more accurately than the approximate method we used. The incorporation of more specific load information, such as data on commercial and multi-family use and the year that the sites began to use water, may also increase the accuracy of the flow assessments, an essential part of the Webler-Brown model. This analysis shows how a detailed retrospective examination of historical measurements made for other purposes can suggest further refinements in the model. While this analysis also supports the exposure model used in previous epidemiologic studies, further analyses are currently underway evaluating the impact of the model's inaccuracies on the risk of breast cancer using data from our prior case-control study [8,9].

## List of Abbreviations

*C*_0*s: *_initial amount of perchloroethylene per unit surface area for pipe segment *s*; DEP: Massachusetts Department of Environmental Protection; *D*_*s: *_Diameter of pipe for pipe segments; EPA: United States Environmental Protection Agency; Ln: Natural logarithm; *L*_*s: *_Length of pipe segments; ND: Not detected; OR: Odds ratio; PC: Point concentration; PCE:  Perchloroethylene or tetrachloroethylene; *Q*_*s*-1_: Magnitude of pipe water flow in pipe segments; *Q*_*x*_: Magnitude of pipe water flow at location *x; r*: Diffusion rate constant; RDD: Relative Delivered Dose; SAG: Suggested No Adverse Response Level; *t*: Time of sampling; *t*_*s*_: Time of pipe installation for pipe segments; VL/AC: Vinyl-lined asbestos-cement.

## Competing interests

Dr. David Ozonoff is Co-editor-in-Chief of *Environmental Health: A Global Access Science Source*. He has recused himself from all decisions involving the acceptance and publication of this manuscript. At the request of the Commonwealth of Massachusetts, Dr. Ozonoff was a witness at the Johns-Manville Corporation bankruptcy hearing in 1980. He has also, on occasion, testified in personal injury cases involving exposure to tetrachloroethylene and trichloroethylene. No such litigation is currently pending. None of the other authors of this study have any competing interests.

## Authors' contributions

LAS carried out a portion of the exposure assessments, conducted DEP file reviews, conducted analyses, and wrote the initial draft of the manuscript. AA conceived the study, participated in its design and coordination, assisted in the analysis, and finalized the manuscript. LG conducted analyses, and helped finalize the manuscript. TW provided technical input to study design, analysis, and modeling. TH provided statistical guidance and review. DO participated in study design, analysis, and manuscript preparation. All authors read and approved the final manuscript.

## Appendix A: Derivation of the point concentration estimate

There are two parts to implementing the Webler Brown model: (1) estimating the water flow in pipes and (2) estimating the movement of PCE from the vinyl liner into the flowing water. The estimated concentration of PCE at location *i *and time *t*, *PC*_*i*_*(t)*, is modeled as the rate that PCE enters pipe water per unit pipe length at upstream position *x*, *F*_*x*_*(t)*, divided by the water flowing at rate *Q*_*x*_, and then integrated along the upstream VL/AC pipe.

(A1) $$PC_{i}(t)=\int_{x=0}^{i}\frac{F_{x}(t)}{Q_{x}}dx$$

To model the PCE leaching rate, *F*_*x*_*(t)*, Webler and Brown [15] began with data generated by Demond [2], who measured the rate of evaporation of PCE from a Piccotex^® ^liner applied to small pieces of aluminum. Webler and Brown fit a first order negative exponent, *e*^-*T/r*^, to these data (*T *is the lapsed time from application of the liner to final PCE measurement, and *r *is the diffusion rate constant) [15]. Incorporating the initial amount of PCE per unit surface area (*C*_0_), Webler and Brown estimated the amount of PCE remaining in the Piccotex^® ^liner at time *t*:

(A2) Cp(t)=C0e−Tr

The flux of PCE from Piccotex was then estimated as the change in PCE per unit surface area over time. Since we are interested in the amount of PCE entering the water, the sign on the flux is positive.

(A3) J(t)=dCp(t)dt=1rC0e−Tr

The resulting leaching rate for PCE per unit length of pipe, *F*_*x*_*(t)*, depends on PCE flux from the liner, *J(t)*, and the surface area across which it moves, *i.e*., for a larger diameter pipe, the incremental contribution at point *x *is greater.

(A4) $$F_{x}(t)=\frac{1}{r}C_{0}e^{\frac{-T}{r}}\pi D_{x}$$

In this equation, *D*_*x *_is the pipe diameter. Thus, *C*_0_*e *^-(*T*/*r*) ^is the amount of initial PCE remaining in the liner after time *T *with first order (exponential) decay. To estimate a point concentration, the leaching rate was divided by the water flow rate (*Q*_*x*_) and integrated along a pipe to the location of interest, *i*.

(A5) $$PC_{i}(t)=\int_{x=0}^{i}\frac{C_{0}e^{\frac{-(T)}{r}}\pi D_{x}}{rQ_{x}}dx$$

This integral is approximated by summing discrete pipe segments we designated to implement the model. Each segment ends at a node, defined as the end point of a segment. The model is developed around these segments (*s*) and nodes and *dx *becomes the pipe segment length (*L*_*s*_). Lapsed time (*T*) is expressed as the time from pipe installation (*t*_*s*_) to the year that the water sample was taken (*t*).

(A6) $$PC_{i}(t)=\sum_{s=1}^{i}\frac{C_{0s}e^{\frac{-(t-t_{s})}{r}}\pi D_{s}L_{s}}{rQ_{s-1}}$$

Water drawn along a segment (*q*_*s*_) was evaluated as removed at the segment node. Thus, the water flow in segment *s *is *Q*_*s*-1_. Equation (A6) gives the estimated micrograms of PCE per liter drinking water at location *i *at time *t *– the quantity that was compared to the measured PCE drinking water concentrations.

The water flow in segment *s*, *Q*_*s*-1_, was estimated as, the flow into a pipe segment minus the water drawn upstream:

(A7) $$Q_{s-1}=Q_{0}-\sum_{z=0}^{s-1}q_{z},$$

The amount of water entering contributing pipe, *Q*_0_, was estimated as the number of homes drawing water along a pipe (*K*_0_) multiplied by the average household water use (*q*). Similarly, the rate at which homes along pipe segment *z *draw water, *q*_*z*_, is the number of homes along segment *z *(*k*_*z*_) multiplied by *q *(*i.e*., if there are 5 homes along segment *z*, *k*_*z *_= 5 and *q*_*z *_= *5q*). *Q*_*s*-1 _is, therefore, estimated by

(A8) $$Q_{s-1}=q(K_{0}-\sum_{z=0}^{s-1}k_{z}).$$

Combining equations (A6) and (A8) yields the PCE point concentration estimate for a specific time (*t*) and location (*i*) in μg/L

(A9) $$PC_{i}(t)=\sum_{s=1}^{i}\frac{C_{0s}e^{\frac{-(t-t_{s})}{r}}\pi D_{s}L_{s}}{rq\left(K_{0}-\sum_{z=0}^{s-1}k_{z}\right)}$$

The parameters in (A9) have the following values:

*C*_0*s *_– The initial amount of PCE per surface area of Piccotex^® ^liner in pipe segment *s*, estimated as 8.56 × 10^7 ^μg/meter^2 ^[15] (Assumes Piccotex^® ^liner application conformed with Johns Manville specifications for the perchloroethylene suspension (30% Piccotex^® ^and 70% PCE), that 6% of PCE remained in the liner at installation, that the liner was uniformly 6.35 × 10^-3 ^meters thick, and that the specific gravity of PCE is 1.624 × 10^9 ^micrograms per cubic meter [2,15].)

*t *– The day of sampling, given as a fraction of a year

*t*_*s *_– The day of pipe installation, estimated as one half of the year of installation (*i.e*., if the water pipe was installed in 1970, *t*_*s *_was estimated as 1970.50 years – approximately July 2, 1970)

*D*_*s *_– Internal water pipe diameter for pipe segment *s *(meters)

*L*_*s *_– Water pipe segment *s *length (meters)

*r *– The PCE diffusion rate constant of 2.25 years [10]

*q *– The average annual household water use was set at 90,000 gallons or 340,687 liters per home per year based on data from the Massachusetts Water Resources Authority 

*K*_0 _– The total number of homes drawing water from contributing VL/AC pipe

*k*_*z *_– The number of homes drawing water along water pipe segment *z*
